# Impact of correlated information on pioneering decisions

**DOI:** 10.1103/physrevresearch.5.033020

**Published:** 2023-07-10

**Authors:** Megan Stickler, William Ott, Zachary P. Kilpatrick, Krešimir Josić, Bhargav R. Karamched

**Affiliations:** 1 Department of Mathematics, University of Houston, Houston, Texas 77004, USA; 2 Department of Applied Mathematics, University of Colorado Boulder, Boulder, Colorado 80309, USA; 3 Department of Biology and Biochemistry, University of Houston, Houston, Texas 77004, USA; 4 Department of Mathematics, Florida State University, Tallahassee, Florida 32306, USA; 5 Institute of Molecular Biophysics, Florida State University, Tallahassee, Florida 32306, USA; 6 Program in Neuroscience, Florida State University, Tallahassee, Florida 32306, USA

## Abstract

Normative models are often used to describe how humans and animals make decisions. These models treat deliberation as the accumulation of uncertain evidence that terminates with a commitment to a choice. When extended to social groups, such models often assume that individuals make independent observations. However, individuals typically gather evidence from common sources, and their observations are rarely independent. Here we ask: For a group of ideal observers who do not exchange information, what is the impact of correlated evidence on decision accuracy? We show that even when agents are identical, correlated evidence causes decision accuracy to depend on temporal decision order. The first decider is less accurate than a lone observer, and early deciders are less accurate than late deciders. These phenomena occur despite the fact that the rational observers use the same decision criterion, so they are equally confident in their decisions. We analyze discrete and macroscopic evidence-gathering models to explain why the first decider is less accurate than a lone observer when evidence is correlated. Pooling the decisions of early deciders using a majority rule does not rescue accuracy results in only a modest accuracy gain. Although we analyze an idealized model, we believe that our analysis offers insights that do not depend on exactly how groups integrate evidence and form decisions.

## I. INTRODUCTION

Most organisms and many computational algorithms make decisions based on a sequence of noisy observations of the environment [1]. Normative models that describe how evidence should be integrated to make the best choice are central to our understanding of such decisions [2]. When an observer needs to choose between alternatives, accumulating evidence refines their perceived probability of the truth of each alternative. Decision policies often prescribe a threshold on the accumulated evidence in order to balance the speed and accuracy of decisions [3,4]. These theories have been developed and validated over decades in experiments with humans and other animals [5–9]. However, most previous work was focused on individual decision makers, and less is known about groups of observers who make choices based on streams of evidence [10,11].

Each member of a social group often needs to choose between the same alternatives based on a combination of correlated and independent observations [12]. For instance, when deciding whom to vote for, two individuals may see some of the same media coverage, but each may also read opinion pieces that the other does not [13]. Conspecifics deciding where to forage are likely to rely on some of the same cues but can also learn from distinct experiences [14]. Traders may have access to private information but often track the same aggregate market indices and reports to decide what stocks to buy and sell, and the processes governing the valuation of distinct commodities are known to be correlated [15]. Thus, even in the absence of direct communication, the measurements individuals in a group use to make decisions are generally *imperfectly* correlated.

**FIG. 1.**
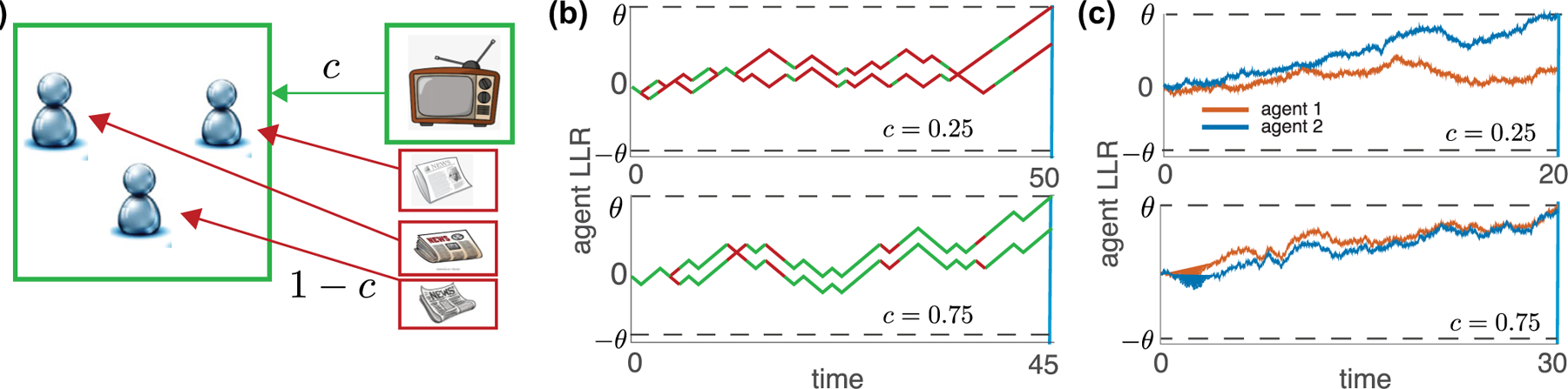
Agents receiving partially correlated evidence. (a) Agents make a sequence of measurements to decide between alternatives. At each time step agents all make the same observation with probability c and independent observations with probability 1−c. (b) Representative trajectories of the log-likelihood ratios (LLRs) computed at discrete times [Eq. (2)] for c=0.25 and c=0.75 and two agents. Green segments correspond to increments due to common observations, and red segments arise from independent observations. An agent commits to a decision $\left(\pm \right)$ when the computed LLR crosses ±θ. (c) Analogous trajectories generated from simulations of the limiting drift-diffusion model [Eq. (3)].

Here we assess the impact of such correlated measurements on the accuracy of individual decisions within groups of agents who do not share information (see Fig. 1). When identical, rational, unbiased agents make *independent* observations the probability of a correct decision is independent of the order or the time at which the decision is made [16,17]. However, when such agents makes correlated measurements, early deciders tend to make decisions based on misleading observations, and their choices are less accurate than those of later deciders by as much as 20%. The order of a decision can therefore determine its accuracy, despite each agent subjectively believing their decision is based on the same amount of evidence, and thus as accurate as that of anyone else. Yet an outsider who observes the order in which decisions are made knows that early decisions are less likely to be correct than later ones. We analytically show why this is the case in tractable examples and provide an intuitive argument explaining why the same holds more generally. Our analysis demonstrates why this difference in accuracy depends on how strongly evidence is correlated and on the size of the population. We also show that pooling early decisions does not always help, but weighting individual decisions according to their order can produce better results.

## II. MODEL

We consider a community of N agents who accumulate evidence to decide between two states, or hypotheses, $H^{+}$ or $H^{-}$. Each agent accumulates evidence (observations) to decide between the two hypotheses. Agents are rational (Bayesian) and compute the probability that either hypothesis holds based on all evidence they accrue. Each makes a decision once the log-likelihood ratio (LLR) of the conditional probabilities between the two hypotheses, given all the accumulated observations, crosses one of two predetermined decision thresholds [2,18]. For simplicity, we assume that the observations the agents make are statistically identical and that they use the same decision policy.

### A. Independent evidence accumulation

The problem of a single agent integrating evidence to decide between two options has been thoroughly studied [2,10,18–21]. In the simplest setting, an agent makes a sequence of noisy observations (measurements), $\xi_{1:t}$, with $\xi_{i}\in \text{Ξ}$ for $i\in \left\{1,\ldots ,t\right\}$, where Ξ⊂ℝ. The observations, $\xi_{i}$, are independent and identically distributed, conditioned on the true state, $H\in \left\{H^{+},H^{-}\right\}$,

$$P\left(\xi_{1:t}\left|H^{\pm }\right.\right)=\prod_{i=1}^{t}P\left(\xi_{i}\left|H^{\pm }\right.\right)=\prod_{i=1}^{t}f_{\pm }\left(\xi_{i}\right).$$

Here the conditional probability of each measurement is given by the probability mass functions, $f_{\pm }\left(\xi \right):=P\left(\xi \left|H^{\pm }\right.\right)$, when the conditional probability distributions are discrete, or by density functions when they are absolutely continuous. Observations, $\xi_{i}$, are drawn from the same set, Ξ, in either state $H^{\pm }$, and the two states are distinguished by the differences in the conditional probabilities of making certain measurements. See Appendix A for details on how the restriction $\text{Ξ}=\left\{\xi^{+},\xi^{-}\right\}$ can confine beliefs to evolving on the integer lattice.

To compute the probability of the two choices, given all observations, $P\left(H^{\pm }\left|\xi_{1:t}\right.\right)$, an ideal observer uses Bayes’ rule. For simplicity, we assume that the agent knows the measurement distributions, $f_{\pm }\left(\xi \right)$, and knows that both environmental states are equally likely, and hence uses a flat prior, $P\left(H^{+}\right)=P\left(H^{-}\right)=1/2$. The log-likelihood ratio (LLR) of the two states at time t is then

(1) $$y_{t}:=\log\left(\frac{P\left(H^{+}\left|\xi_{1:t}\right.\right)}{P\left(H^{-}\left|\xi_{1:t}\right.\right)}\right)=\sum_{s=1}^{t}\text{LLR}\left(\xi_{s}\right)=y_{t-1}+\text{LLR}\left(\xi_{t}\right),$$

where $\text{LLR}\left(\cdot \right)\equiv \log\frac{P\left(\cdot \left|H^{+}\right.\right)}{P\left(\cdot \left|H^{-}\right.\right)}$. We also refer to $y_{t}$ as the *belief* of the agent at time t. The magnitude of the LLR can be viewed as the information an agent has gathered in support of a hypothesis, while its sign describes the choice preference ($H^{+}$ or $H^{-}$) of the agent. The flat prior implies $y_{0}=\log\frac{P\left(H^{+}\right)}{P\left(H^{-}\right)}=\log\frac{1/2}{1/2}=0$.

The optimality of the sequential probability ratio test [18] implies that an individual agent best manages speed and accuracy by waiting to decide until their belief reaches or crosses above (below) an upper (lower) threshold $\theta_{+}>0\left(\theta_{-}<0\right)$. Thus, an ideal agent continues making observations while $\theta_{-}<y_{t}<\theta_{+}$ and makes a decision after acquiring sufficient evidence, choosing $H^{+}\left(H^{-}\right)$ once $y_{t}\;\;⩾\;\;\theta_{+}\left(y_{t}\;\;⩽\;\;\theta_{-}\right)$. We have analyzed a generalization of this model to social networks both small [22] and large [17], where each agent accrues independent information according to Eq. (1) and shares their decision state with some or all other agents in the group. These models of normative information exchange based on neighbors’ decisions build on previous work on normative confidence weighting for majority rules [23–26], locally optimal Bayesian integration on sparse graphs [27], the impact of common observations [16], and nonnormative decision sharing [28].

### B. Accumulation of correlated measurements

We analyze the impact of correlated information on the accuracy of the decisions of a community of N independent and isolated agents. At each time step, t, every agent, i, makes an observation (measurement), $\xi_{t}^{i}\in \text{Ξ}$, and updates their private belief, $y_{t}^{i}$, according to Eq. (1). However, an individual agent does not know whether others have made decisions or what those decisions were, in contrast to social network models studied in the past [17,22–32]. This could be a model of a sample of voters, each of whom does not know the others, or traders deciding to buy or sell a stock without tipping their hand.

To model correlated measurements, we assume that on each time step all agents make an identical observation with probability c. An identical observation means that $\xi_{t}^{i}=\xi_{t}$ for all agents, i=1,…,N, where $\xi_{t}$ is a single sample from the measurement distribution, $f_{\pm }\left(\xi \right)$. With probability 1−c agents make independent observations during a time step, and the N measurements, $\xi_{t}^{i}$, are sampled independently from the distribution $f_{\pm }\left(\xi \right)$. This is equivalent to having N *private*, independent sources of evidence, each accessible to a single agent, and one *common* evidence source accessible to all agents (see the Discussion for less restrictive assumptions). Therefore, the belief of each agent evolves according to

(2) $$\begin{array}{l} y_{t}^{i}=y_{t-1}^{i}+\left(1-\chi_{t}\right)\text{LLR}\left(\xi_{t}^{i}\right)+\chi_{t}\text{LLR}\left(\xi_{t}\right), \end{array}$$

where $\chi_{t}$ are i.i.d. Bernoulli random variables each with parameter c. When c=1 agents make only common observations, and when c=0 agents make only independent observations. As c increases from zero, each observation is more likely to be common, and the overall evidence becomes more correlated.

Each agent makes observations until their belief, $y_{t}^{i}$, reaches one of the thresholds, $\theta_{\pm }$, at which point they make the corresponding decision, $H^{\pm }$. For simplicity we henceforth assume the thresholds are symmetric about zero, i.e., $\theta_{\pm }=\pm \theta$, with θ>0. We denote the decision time of agent i by $T_{i}$, and assume that decisions are immutable. Thus, decision times are uniquely defined, and only undecided agents continue to make observations.

Importantly, each agent is isolated and *does not* observe others’ decisions or their decision state (decided or undecided), in contrast with [17,22]. Agents do not know whether an observation is common or private, and each uses the evidence they have collected to make the best possible decision based on their belief (LLR) given by Eq. (2).

We ask how the accuracy of an agent’s decision depends on the order in which the decision is made. In particular, how accurate is the first decider? If multiple agents make a decision at first-decision time, the “first” decider is chosen randomly with equal probability from that group. The probability of a correct first decision then equals the probability that this first decider makes the correct choice, i.e., that the belief of the first decider reaches the threshold, ±θ, whose sign agrees with that of the true environmental state, $H^{\pm }$. We briefly discuss other ways of defining a first decision in Appendix B.

### C. Scaling limit of correlated evidence accumulation

Computing decision accuracy and the distribution of decision times reduces to a first-passage problem [2]. Often it is easier to solve such problems in the scaling limit, thus avoiding the combinatorial challenges common in discrete problems [33]. By invoking the Donsker Invariance Principle [34], in the limit of infinitely many infinitesimally informative measurements we obtain the macroscopic version of Eq. (2), often referred to as a *drift-diffusion equation*:

(3) $$dy_{i}=\pm \mu \;dt+\sqrt{2\left(1-c\right)\mu }\;dW_{i}+\sqrt{2c\mu }\;dW_{c}.$$

Here $y_{i}\left(t\right)$ is the limit of the LLR of agent i and μ scales both the drift and diffusion terms. See Appendix C for a derivation of Eq. (3), verification of agreement with the discrete model, and definition of μ, which is proportional to the square of the signal-to-noise ratio of the sample distribution. The sign of the drift agrees with the sign of the environmental state, $H^{\pm }$. The Wiener processes, $W_{i}\left(t\right)$ and $W_{c}\left(t\right)$, capture the variability of belief increments due to independent and common observations, respectively. Thus, the belief of each observer, $y_{i}\left(t\right)$, evolves according to a drift-diffusion model [2] with a combination of independent and correlated noise sources. This model has been analyzed previously [16,35], but we are not aware of a previous derivation from the normative model (see Discussion).

## III. RESULTS

**FIG. 2.**
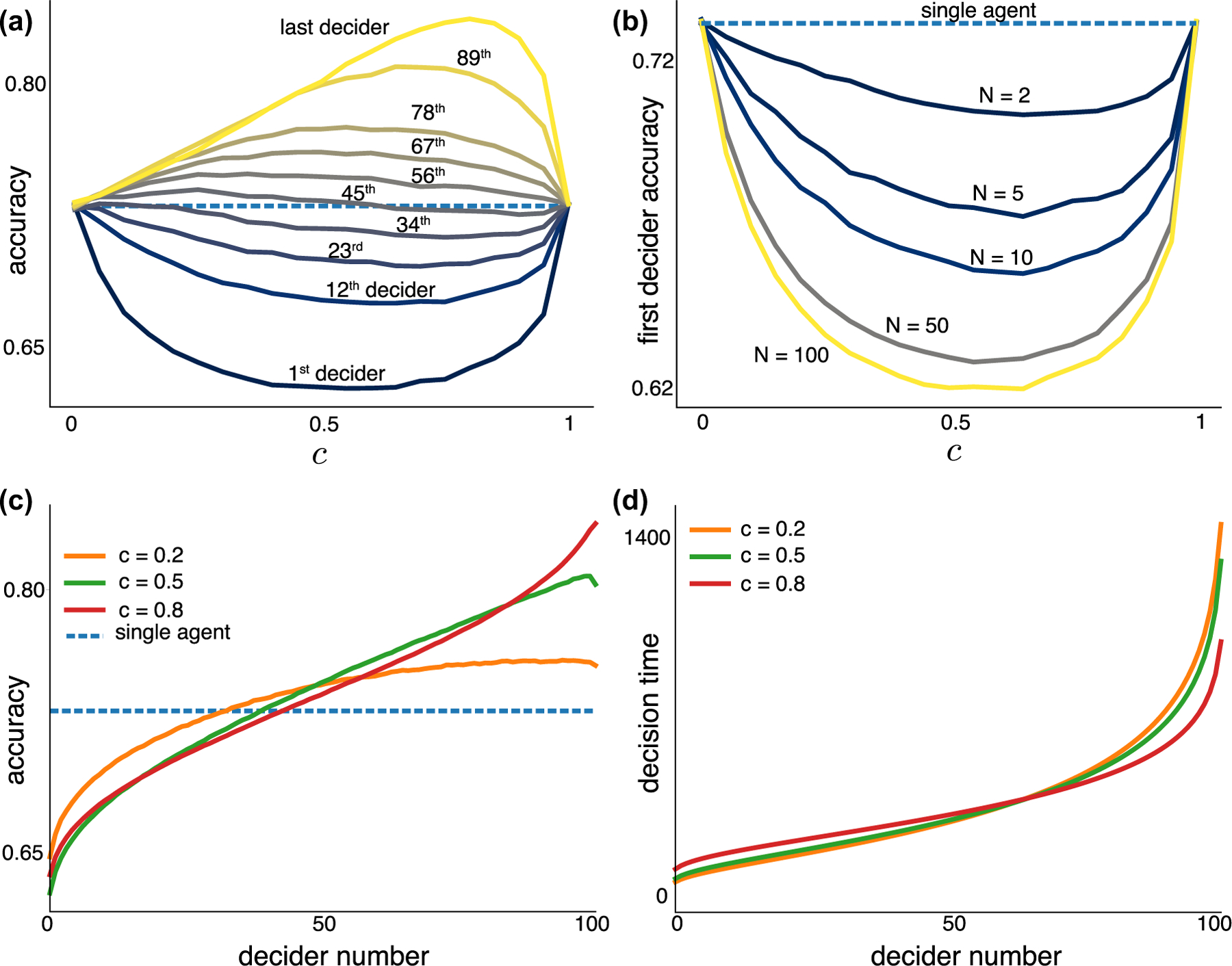
Impact of the probability of making a common observation, c, on decider accuracy and timing. (a) The probability of a correct decision increases with the order in which the decision is made (solid lines) and varies nonmonotonically with c. The average accuracy computed over all deciders (dashed line) equals the accuracy of a randomly chosen agent and is constant with c. N=100. (b) The accuracy of the first decider varies nonmonotonically with c, possessing an internal minimum $0<c_{\min}<1$. As N increases, the lowest accuracy decreases. (c) The accuracy of each of N=100 deciders increases with decision order almost monotonically, so the first (last) decider is less (more) accurate than a lone decider for c≠0, 1. (d) The time of the decision of N=100 agents as a function of order is approximately invariant to changes in the probability of common evidence, c. We used the discrete LLR model Eq. (2) with θ=10 and binary likelihood functions $f_{\pm }$ as described in Appendix A. Specifically we chose $f_{\pm }$ so that the update size is ±0.05.

We first asked how correlated evidence impacts the accuracy of decisions within a group of rational, identical agents. The probability that a randomly selected agent in the group makes a correct choice does not depend on the number of other agents or on how strongly the evidence is correlated. However, for all 0<c<1, the probability that the *first* decider in the group is correct is *smaller* than the probability that a lone observer is correct, reaching an internal minimum [Fig. 2(a)].

Since every individual agent’s perception of the correct hypothesis and decision process are described by the same stochastic process, each agent has the same subjective estimate that their choice is correct, $\left(1/\left(1+e^{-\theta }\right.\right)$ [2,36]. Indeed, this is the probability that a randomly chosen agent makes a correct decision. However, the first agent to make a decision is less likely to make a correct choice than all other agents in a group, and this probability decreases with the number of agents in the community [Fig. 2(b)]. Furthermore, decider accuracy increases almost monotonically with the order of the decision [Fig. 2(c)]. Thus, someone observing the order in which decisions are made should trust later decisions more than early ones. Decision times of distinct agents get closer as common observations become more probable [Fig. 2(d)], since the observers’ beliefs evolve more synchronously.

The decreased accuracy of the first decider for 0<c<1 relative to single-decider accuracy is not a trivial consequence of early deciders spending less time accumulating evidence. If this were the case, the first decider would be less accurate than later ones when c=0. But when all observations are independent, the probability of a correct decision is independent of the order in which the decision is made and is determined by the decision threshold. However, a common initial bias can also lead to accuracies that depend on decision order, even when measurements are independent (see Discussion as well as [22,37]). Moreover, as c is increased from 0 to approximately 0.5, the average time to the first decision increases, but the average accuracy of this decision decreases. We next provide an explanation of this observation.

### A. An intuitive explanation for the decrease in first decision accuracy

Why do common observations lead to less accurate first decisions? At the time of the first decision, the remaining undecided agents have likely made independent observations that counter the common observations that often contribute to the first decider’s choice. Indeed, if these independent observations aligned with this choice and the common evidence, the other agents would likely have already made a decision as well. For small c, little information is gained from common evidence, and not much independently gathered evidence is needed to counter it. As c increases, common evidence more often drives the first decision, so we expect a substantial fraction of the independent evidence collected by an undecided agent will often counter the common evidence. However, when c is large, most evidence is common, and fewer observations are independent, leaving less time for strong, contrary independent observations. Thus, at a critical value of c, the average total independent evidence obtained by undecided agents countering common observations reaches a maximum. The probability of a correct first decision is smallest at this critical value. In the next subsection, we sharpen this argument by showing independent observations made by undecided agents that favor the correct decision are stronger when the first decider makes an incorrect choice than in the opposite case.

### B. Reduction of the log-likelihood ratio of the first decider

We next show mathematically why the first decider’s choice is less accurate than that of a randomly chosen agent selected with equal probability from all agents in the group prior to evidence accumulation. To do so, we write the log-likelihood ratio (LLR) associated with the probability the first decider makes the correct choice as a sum of two terms: One term is the LLR of a randomly selected agent at decision time, while the second incorporates the condition that this agent is the first decider. We show that the first term’s magnitude equals θ, while the second term is negative for 0<c<1. Thus, the information obtained by undecided agents reduces the probability of a correct first decision. We begin by considering a pair of agents and obtain expressions for the sum of LLR terms in the case of beliefs evolving on a lattice. We then extend this calculation to an arbitrary number of agents.

#### 1. Pair of agents in discrete time

We randomly number the agents using indices j=1,2, and let FD be the index of the first decider. Let $T_{j}$ be the time of the decision of agent j, and denote the decision of agent j by $d_{j}\in \left\{H^{+},H^{-}\right\}$, so that $y_{j}\left(T_{j}\right)=\pm \theta$ and $\left|y_{j}\left(t\right)\right|<\theta$ when $0\;\;⩽\;\;t<T_{j}$. Let $T=\min\left(T_{1},T_{2}\right)$ denote the time of the first decision. We assume the first decider chooses $H^{+}\left(d_{FD}=H^{+}\right)$ without loss of generality (WLOG), and write the conditional probability $P^{\pm }\left(d_{FD}=H^{+}\right):=P\left(d_{FD}=H^{+}\left|H^{\pm }\right.\right)$ as

(4) $$\begin{array}{l} P^{\pm }\left(d_{FD}=H^{+}\right)=\sum_{j=1}^{2}P^{\pm }\left(d_{j}=H^{+},FD=j\right)\\ \;\;\;\;\;\;\;\;\;\;\;\;\;\;=\sum_{j=1}^{2}P^{\pm }\left(FD=j\left|d_{j}=H^{+}\right.\right)P^{\pm }\left(d_{j}=H^{+}\right)\\ \;\;\;\;\;\;\;\;\;\;\;\;=2P^{\pm }\left(d_{1}=H^{+}\right)P^{\pm }\left(FD=1\left|d_{1}=H^{+}\right.\right), \end{array}$$

where the final line follows from the exchange symmetry between the two agents. The first term in Eq. (4) is the $P^{\pm }$ probability that a randomly chosen agent (here agent 1, WLOG) selects $H^{+}$, depending only on agent 1’s observations. The second term is the $P^{\pm }$ probability that, conditioned on choosing $H^{+}$, agent 1 is also the first to decide, which depends on information gathered by agent 2.

The second term on the right side of Eq. (4) can be rewritten as a sum over $T_{1}$, $T_{2}\in \mathbb{N}$ and then simplified by noting that FD=1 with certainty if $T_{1}<T_{2}$ and with probability 1/2 if $T_{1}=T_{2}$:

$$\begin{array}{l} P^{\pm }\left(d_{FD}=H^{+}\right)=2P^{\pm }\left(d_{1}=H^{+}\right)\sum_{t_{1}\in \mathbb{N}}\sum_{t_{2}\in \mathbb{N}}P^{\pm }\left(FD=1\left|T_{1}=t_{1},T_{2}=t_{2},d_{1}=H^{+}\right.\right)P^{\pm }\left(T_{1}=t_{1},T_{2}=t_{2}\left|d_{1}=H^{+}\right.\right),\\ \;\;\;\;\;\;\;\;\;\;\;\;\;\;=2P^{\pm }\left(d_{1}=H^{+}\right)\sum_{t_{1}\in \mathbb{N}}\left[\frac{1}{2}P^{\pm }\left(t_{1}=T_{1}=T_{2}\left|d_{1}=H^{+}\right.\right)+P^{\pm }\left(t_{1}=T_{1}<T_{2}\left|d_{1}=H^{+}\right.\right)\right]. \end{array}$$

Using Eq. (4) we can thus write the corresponding LLR of the first decider at the time of their decision as

$$\text{LLR}\left(d_{FD}=H^{+}\right)=\log\frac{P^{+}\left(d_{FD}=H^{+}\right)}{P^{-}\left(d_{FD}=H^{+}\right)}=\text{LLR}\left(d_{1}=H^{+}\right)+\text{LLR}\left(FD=1\left|d_{1}=H^{+}\right.\right).$$

The first term in this sum is the LLR of the decision a randomly chosen agent (taken here to be agent 1 WLOG), $\text{LLR}\left(d_{1}=H^{+}\right)=\theta$. The second term is given by

(5) $$\text{LLR}\left(FD=1\left|d_{1}=H^{+}\right.\right)=\log\frac{\sum_{t_{1}\in \mathbb{N}}\left[\frac{1}{2}P^{+}\left(t_{1}=T_{1}=T_{2}\left|d_{1}=H^{+}\right.\right)+P^{+}\left(t_{1}=T_{1}<T_{2}\left|d_{1}=H^{+}\right.\right)\right]}{\sum_{t_{1}\in \mathbb{N}}\left[\frac{1}{2}P^{-}\left(t_{1}=T_{1}=T_{2}\left|d_{1}=H^{+}\right.\right)+P^{-}\left(t_{1}=T_{1}<T_{2}\left|d_{1}=H^{+}\right.\right)\right]}.$$

Now, if agent 1 has made an incorrect decision, one inconsistent with the true hypothesis, both this agent’s common and independent observations are likely to support the incorrect decision. But, by assumption, any randomly sampled observation is more likely to be consistent with the true than the wrong hypothesis. Thus, the independent observations of agent 2 are likely to point to the correct hypothesis, conflicting with the common observations supporting the incorrect decision of agent 1. As a result, agent 2 more likely decides after $T_{1}$ when agent 1’s choice is wrong than when it is correct. This argument shows that we expect

(6) $$\sum_{t_{1}\in \mathbb{N}}\left[\frac{1}{2}P^{+}\left(t_{1}=T_{1}=T_{2}\left|d_{1}=H^{+}\right.\right)+P^{+}\left(t_{1}=T_{1}<T_{2}\left|d_{1}=H^{+}\right.\right)\right]\;\;\;<\sum_{t_{1}\in \mathbb{N}}\left[\frac{1}{2}P^{-}\left(t_{1}=T_{1}=T_{2}\left|d_{1}=H^{+}\right.\right)+P^{-}\left(t_{1}=T_{1}<T_{2}\left|d_{1}=H^{+}\right.\right)\right]$$

for 0<c<1, so that Eq. (5) implies $\text{LLR}\left(FD=1\left|d_{1}=H^{+}\right.\right)<0$ for such values of c. As a result, $\text{LLR}\left(d_{FD}=H^{+}\right)<\text{LLR}\left(d_{i}=H^{+}\right)=\theta$ for i=1,2 and 0<c<1, so the first decider makes a correct choice less often than an agent chosen at random. This argument can be extended to N>2 agents in most cases, demonstrating an increased probability and volume of contrary evidence in more remaining undecided agents, causing a larger drop in the first decider’s accuracy (see Appendix C).

Moreover, as c increases, so does the fraction of wrong common observations that can be countered by correct independent observations of agent 2. This initially increases the likelihood that agent 2 remains undecided following incorrect decisions by agent 1. But if c is high, most observations are common, and agent 2 makes few independent observations. Thus, as c approaches 1 the agents’ beliefs tend to evolve more synchronously, and the difference between the left and right sides of inequality (6) decreases. This tension between the increase, with c, in the fraction of wrong common observations that are likely to be counteracted, and the decrease in the fraction of correct independent observations that can counteract them causes Eq. (5) to achieve an internal minimum, $0<c_{\min}<1$.

**FIG. 3.**
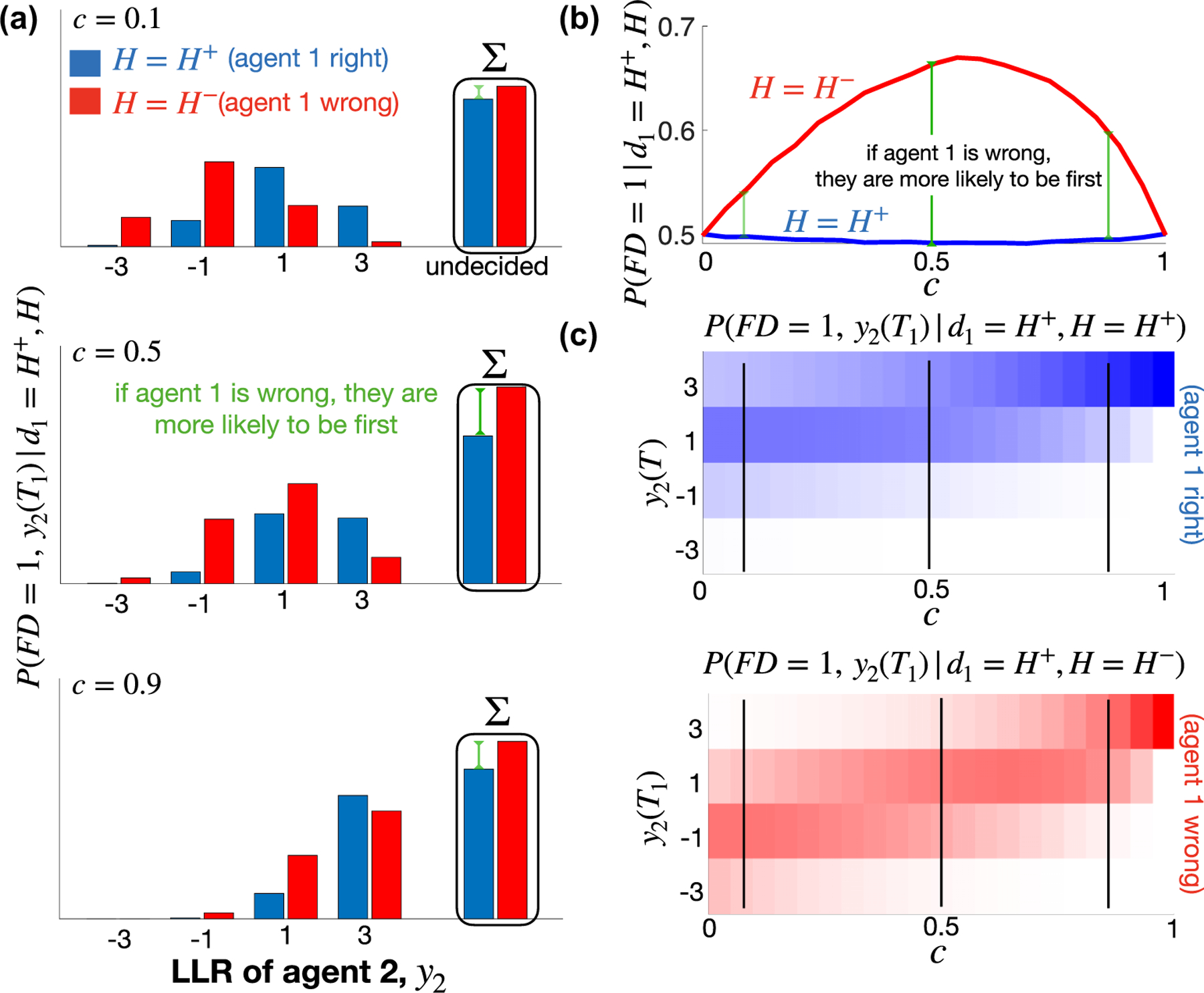
When evidence is partially correlated, a randomly selected agent is more likely to be the first decider if they are wrong. (a) Joint distribution of the probability that agent 1 decides first $\left(FD=1\right)$ and the belief of agent 2 at that decision time, $y_{2}=y_{2}\left(T_{1}\right)$, conditioned on agent 1 being right ($d_{1}=H^{+}=H$, blue) or wrong ($d_{1}=H^{+}\neq H$, red). When 0<c<1, the accuracy of the first decider is strictly below that of a randomly selected agent (here agent 1, WLOG) because of inequality (6). When c is small, $P^{+}\left(FD=1\left|d_{1}=H^{+}\right.\right)$ nearly equals $P^{-}\left(FD=1\left|d_{1}=H^{+}\right.\right)$ (difference indicated by green line), since the joint distributions are approximately reflections of one another, i.e., $P^{+}\left(FD=1,y_{2}\left(T_{1}\right)\left|d_{1}=H^{+}\right.\right)\approx P^{-}\left(FD=1,-y_{2}\left(T_{1}\right)\left|d_{1}=H^{+}\right.\right)$, with equality holding when c=0. As c increases, the difference $P^{-}\left(FD=1\left|d_{1}=H^{+}\right.\right)-P^{+}\left(FD=1\left|d_{1}=H^{+}\right.\right)$ first grows $\left(c=0.5\right)$ and then shrinks $\left(c=0.9\right)$, as both terms converge to 1/2 as c→1. As discussed in Appendix A, each observation changes an agent’s belief, $y_{j}$, by ±1; e.g., when FD=1 and $y_{1}\left(T_{1}\right)=\pm 3$, then $y_{2}\left(T_{1}\right)$ is also an odd integer. (b) The probability that agent 1 decides first (conditioned on $d_{1}=H^{+}$ and $H=H^{-}$) as a function of c peaks around c=0.5. (c) Colormap of the joint distributions from (a) as functions of c. Here we used the discrete LLR model (2) with θ=3 and binary likelihood functions $f_{\pm }$ as described in Appendix A. $f_{\pm }$ are chosen so the update size is ±1.

Numerical experiments support this explanation. Figure 3 illustrates the case of two agents, each with decision threshold magnitude θ=3. As our argument predicts, $P^{+}\left(FD=1\left|d_{1}=H^{+}\right.\right)<P^{-}\left(FD=1\left|d_{1}=H^{+}\right.\right)$ for all 0<c<1 [Fig. 3(b)]. Further, the difference $P^{-}\left(FD=1\left|d_{1}=H^{+}\right.\right)-P^{+}\left(FD=1\left|d_{1}=H^{+}\right.\right)$ first grows and then shrinks as c increases, due mainly to the unimodalilty of the conditional probability that agent 1 decides first when their choice is wrong, $P^{-}\left(FD=1\left|d_{1}=H^{+}\right.\right)$. Looking at the joint conditional probabilities of FD=1 and the belief of agent 2 at the time of the first decision, $P^{+}\left(FD=1,y_{2}\left(T\right)\left|d_{1}=H^{+}\right.\right)$ and $P^{-}\left(FD=1,y_{2}\left(T\right)\left|d_{1}=H^{+}\right.\right)$ helps illuminate the situation. Figure 3(a) shows these joint distributions for representative values of c with θ=3. The distribution of beliefs, $y_{2}$, concentrates more on values $y_{2}\left(T\right)=\pm 1$, away from the thresholds, when $H=H^{-}$ than when $H=H^{+}$ for intermediate values of c [Fig. 3(c)].

#### 2. Two agents with decision threshold magnitude θ=2

We now discuss the case where θ=2, allowing us to compute exact expressions for Eq. (5), since two measurements are sufficient for belief magnitude to reach the bound. As in Appendix A, we assume there can only be two measurement values $\left(\xi^{\pm }\right)$, and $f_{\pm }\left(\xi^{\pm }\right)=p_{+}=e/\left(e+1\right)$ and $f_{\pm }\left(\xi^{\mp }\right)=1-p_{+}\equiv p_{-}$, so beliefs are restricted to the integer lattice. Setting thresholds to ±θ=±2, the belief of any undecided agent, i, must equal $y_{t}^{i}=\pm 1$, at any odd time, and $y_{t}^{i}=0$ at any even time. Thus, the stochastic process governing the evidence accumulation of undecided agents resets to 0 (renews) every two time steps. If T is the time of the first decision, then

$$P\left(d_{FD}=H^{+},T=t\left|T>t-2\right.\right)=P\left(d_{FD}=H^{\pm },T=2\right)$$

for all even t>0, since if T>t−2 then at time t−2 both agents must have been undecided with beliefs $y_{t-2}^{i}=0$.

We now enumerate and sum the probabilities of all cases in which agent 1 (not necessarily the first decider) makes decision $d_{1}=H^{+}$ under either condition, $H=H^{\pm }$. There are four ways for the two agents to make a simultaneous decision: If $d_{1}=H^{+}$, agent 2 can make the same decision $\left(d_{2}=H^{+}\right)$ given zero, one, or two independent measurements, or the opposite choice $\left(d_{2}=H^{-}\right)$, if they made two independent measurements. Therefore,

$$\begin{array}{l} P^{\pm }\left(T_{1}=T_{2}=t\left|d_{1}=H^{+},T>t-2\right.\right)\\ \;\;\;\;\;\;=c^{2}+2c\left(1-c\right)p_{\pm }+{\left(1-c\right)}^{2}\left(p_{+}^{2}+p_{-}^{2}\right). \end{array}$$

The second agent may remain undecided at the time of the first agent’s decision if they made one independent measurement that conflicts with the first agent’s decision, or two independent measurements that conflict with each other:

(7) $$\begin{array}{l} P^{\pm }\left(T_{1}=t<T_{2}\left|d_{1}=H^{+},T>t-2\right.\right)\\ \;\;\;\;\;\;=2c\left(1-c\right)p_{\mp }+2{\left(1-c\right)}^{2}p_{+}p_{-}. \end{array}$$

Now let m∈ℕ and $t_{1}=2m$. Referring to the sums in Eq. (5), we have

$$\begin{array}{l} P^{\pm }\left(t_{1}=T_{1}=T_{2}\left|d_{1}=H^{+}\right.\right)\\ \;\;\;\;\;\;=P^{\pm }\left(t_{1}=T_{1}=T_{2}\left|d_{1}=H^{+},T>t_{1}-2\right.\right)\\ \;\;\;\;\;\;\;\;\;\;\times P^{\pm }\left(T>t_{1}-2\left|d_{1}=H^{+}\right.\right)\\ \;\;\;\;\;\;=P^{\pm }\left(t_{1}=T_{1}=T_{2}\left|d_{1}=H^{+},T>t_{1}-2\right.\right)P\left(T>t_{1}-2\right)\\ \;\;\;\;\;\;=P^{\pm }\left(t_{1}=T_{1}=T_{2}\left|d_{1}=H^{+},T>t_{1}-2\right.\right){\left[P\left(T>2\right)\right]}^{m-1}. \end{array}$$

A similar calculation gives

$$\begin{array}{l} P^{\pm }\left(t_{1}=T_{1}<T_{2}\left|d_{1}=H^{+}\right.\right)=P^{\pm }(t_{1}=T_{1}<T_{2}\left|d_{1}\right.\\ \;\;\;\;\;=H^{+},T>t_{1}-2){\left[P\left(T>2\right)\right]}^{m-1}. \end{array}$$

We factor common terms out of the sums in Eq. (5) and cancel sums over factors of $P{\left(T>2\right)}^{m-1}$ in the numerator and denominator to obtain an explicit form of Eq. (5),

(8) $$\begin{array}{l} \text{LLR}\left(FD=1\left|d_{1}=H^{+}\right.\right)\\ \;\;\;\;\;\;\;\;=\log\frac{\left[c^{2}+2c\left(1-c\right)\left(1+p_{-}\right)+{\left(1-c\right)}^{2}\left(1+2p_{+}p_{-}\right)\right]}{\left[c^{2}+2c\left(1-c\right)\left(1+p_{+}\right)+{\left(1-c\right)}^{2}\left(1+2p_{+}p_{-}\right)\right]}. \end{array}$$

The numerator and the denominator in this expression differ only in the middle terms, $2c\left(1-c\right)\left(1+p_{-}\right)<2c\left(1-c\right)\left(1+p_{+}\right)$ for 0<c<1, which is the probability that agent 2 makes an independent observation that counters the agents’ common observation, in agreement with our general explanation. As discussed previously, this is more likely when the decision of the first agent (and the common measurement) is wrong.

#### 3. Macroscopic case

Our results for the discrete model extend to agents with continuously evolving beliefs, obtained in the limit of many weak observations (see Appendix C). Agents’ beliefs, $y_{j}\left(t\right)$, each evolve according to Eq. (3) until crossing a threshold ±θ, determining the choice $d_{j}=H^{\pm }$ and decision time $T_{j}\in \left(0,\infty \right)$ for j=1,…,N. Define $T=\left(T_{1},\ldots ,T_{N}\right)\in {\left(0,\infty \right)}^{N}\equiv {\mathbb{R}}_{+}^{N}$. For finite N and c<1, the probability that two agents decide at the same time is zero, so we need not account for simultaneous decisions. By marginalizing over all agents and decision times, we obtain

$$\begin{array}{l} P^{\pm }\left(d_{FD}=H^{+}\right)=NP^{\pm }\left(d_{1}=H^{+}\right)\\ \;\;\;\times \int_{{\mathbb{R}}_{+}^{N}}P^{\pm }\left(FD=1\left|T=t,d_{1}=H^{+}\right.\right)g^{\pm }\left(t\left|d_{1}=H^{+}\right.\right)dt. \end{array}$$

Here $g^{\pm }\left(\cdot \left|d_{1}=H^{+}\right.\right)$ is the conditional probability density function for T, conditioned on the state, $H=H^{+}$, and on the decision $d_{1}=H^{+}$. We have $P^{\pm }\left(FD=1\left|T=t,d_{1}=H^{+}\right.\right)=1$ if $t_{1}=\min_{1⩽i⩽N}t_{i}$ and otherwise this quantity is zero, simplifying the multi-dimensional integral in the preceding expression to an integral over the $t_{1}$ axis and allowing us to write

$$\text{LLR}\left(d_{FD}=H^{+}\right)=\theta +\log\frac{\int_{{\mathbb{R}}_{+}}G^{+}\left(t_{1}\left|d_{1}=H^{+}\right.\right)dt_{1}}{\int_{{\mathbb{R}}_{+}}G^{-}\left(t_{1}\left|d_{1}=H^{+}\right.\right)dt_{1}},$$

where the second term is the log of the ratio of the probabilities that all other agents are undecided at the time at which agent 1 chooses $H^{+}$. Terms in the ratio are obtained by integrating the probability density

$$\begin{array}{l} G^{\pm }\left(t_{1}\left|d_{1}=H^{+}\right.\right)\\ \;\;\;=\int_{{\left(t_{1},\infty \right)}^{N-1}}g^{\pm }\left(t_{1},t_{2},\ldots ,t_{N}\left|d_{1}=H^{+}\right.\right)dt_{2}\cdots dt_{N}, \end{array}$$

across all possible times of the decision of agent 1, given that agent 1 chooses $H^{+}$.

**FIG. 4.**
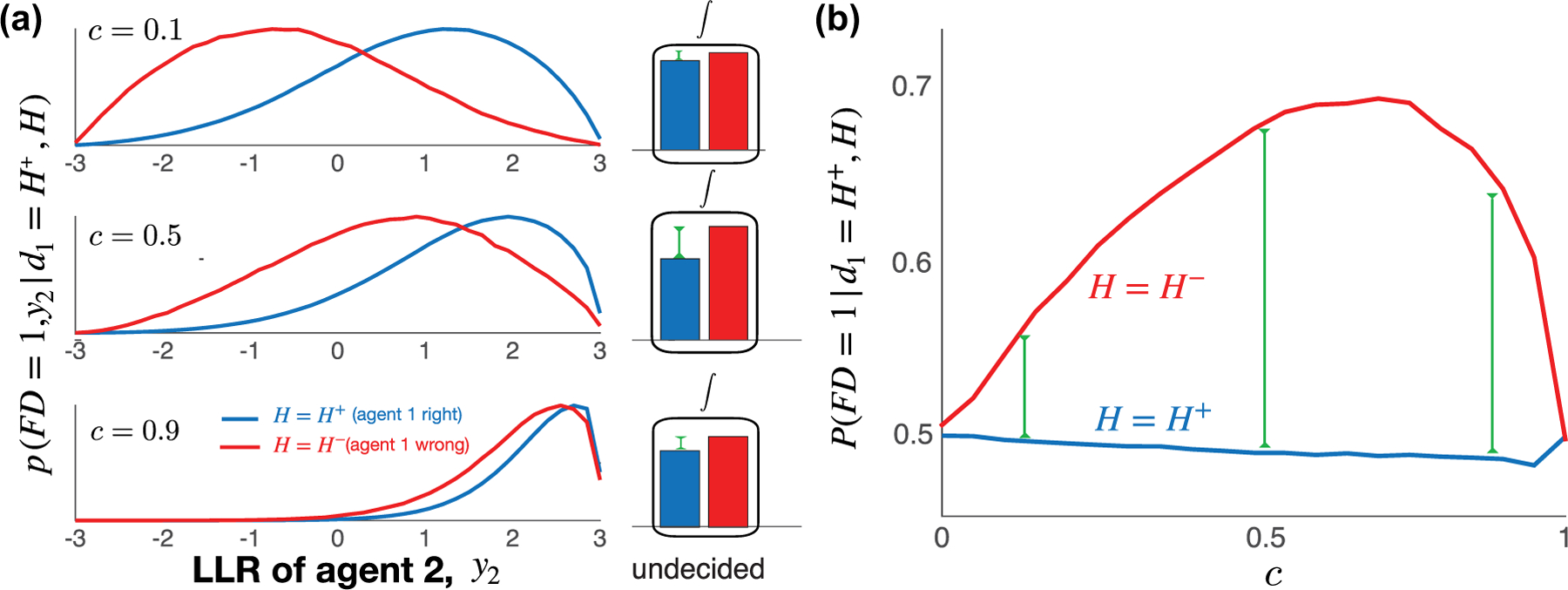
When beliefs evolve continuously and evidence is correlated, a randomly selected agent is again more likely to decide first if they are wrong. (a) As in the discrete model, the densities $p\left(FD=1,y_{2}\left|d_{1}=H^{+},H\right.\right)$ are nearly reflections of one another for small c. By marginalizing over the distribution of beliefs, $y_{2}$, we can obtain the difference $P^{-}\left(FD=1\left|d_{1}=H^{+}\right.\right)-P^{+}\left(FD=1\left|d_{1}=H^{+}\right.\right)$. This difference is small when c is small (red bar minus blue bar). As c increases, this difference first increases and then decreases, the latter because each term in the difference converges to 1/2 as c→1. (b) The unimodal response of first-decider accuracy as c increases is again due to $P^{-}\left(FD=1\left|d_{1}=H^{+}\right.\right)$ obtaining a maximum around c=0.5. We used the macroscopic model (3) with unit drift and variance and threshold θ=3.

When N=2, the nonmonotonicity of the first decider’s accuracy in c is due to the tension between opportunity for contradiction in agent 2’s observations and the decreasing prevalence of independent observations, as c increases. The densities $\frac{d}{dz}P\left(FD=1,y_{2}\left(T\right)⩽z\left|d_{1}=H^{+},H\right.\right)$ are nearly reflections of one another for small c [Fig. 4(a), top left]. Integrating over z, the difference $P^{-}\left(FD=1\left|d_{1}=H^{+}\right.\right)-P^{+}\left(FD=1\left|d_{1}=H^{+}\right.\right)$ is small when c is small [red bar minus blue bar, Fig. 4(a), top center]. For intermediate values of c, the distribution of beliefs of agent 2 is pulled away from the correct threshold when agent 1 decides incorrectly, due to common observations, causing $P^{-}\left(FD=1\left|d_{1}=H^{+}\right.\right)-P^{+}\left(FD=1\left|d_{1}=H^{+}\right.\right)$ to reach a maximum within the intermediate c range. When c is close to 1, both $P^{-}\left(FD=1\left|d_{1}=H^{+}\right.\right)$ and $P^{+}\left(FD=1\left|d_{1}=H^{+}\right.\right)$ converge to 1/2, so the difference converges to zero. Figure 4(b) shows that the unimodal response of first-decider accuracy as c increases occurs because the probability $P^{-}\left(FD=1\left|d_{1}=H^{+}\right.\right)$ of an incorrect agent deciding first increases for small c and then decreases in c (red curve), while $P^{+}\left(FD=1\left|d_{1}=H^{+}\right.\right)$ is approximately insensitive to c (blue curve).

### C. Pooling over early deciders does not rescue accuracy

**FIG. 5.**
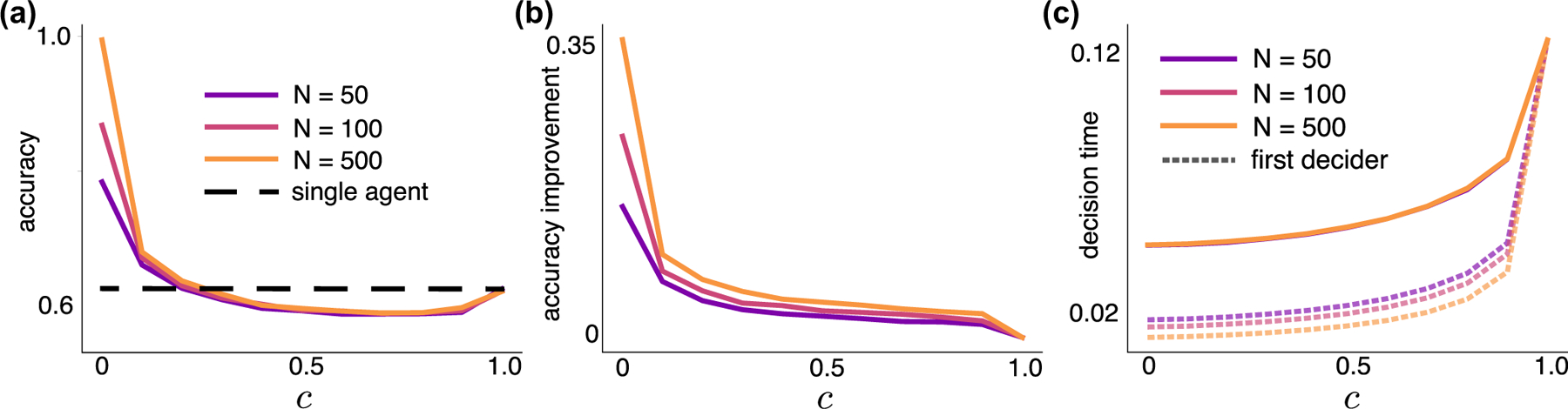
Pooling choices of early deciders using a majority rule mildly improves accuracy compared to the first decision when evidence is correlated. (a) The group’s decision is determined by the majority of the first $N_{pool}$ deciders. For different population sizes, N, the accuracy of the group decision at first decreases as c is increased and can be lower than the accuracy of a single decider in isolation (dashed line). $N_{pool}=0.2N$. (b) Improvement in the accuracy obtained by pooling the first $N_{pool}=0.2N$ decisions compared with the accuracy of the first decision drops substantially even for small values of c and is nearly independent of N. (c) The mean time at which the last decider in the pool makes a decision increases with c (solid curves). Dashed curves give the mean time of the first decision. We used the macroscopic LLR (3) with threshold θ=10.

The “wisdom of crowds” is the idea that collective decision by a group of people is more likely to be correct than the decision of any single member of the group [23,38]. A group’s decision accuracy can be improved when individuals exchange information preceding their final decisions or when the group decision is determined by the majority of individual choices [17,23,27,39,40]. However, this improvement can be diminished, and individuals can even outperform crowds when biases in individual decisions are not accounted for when forming the group decision [41,42]. Applying a majority rule to an initial pool of early deciders, we show that even modest correlations in information can cause this pool to make less accurate choices than a randomly selected agent and only slightly improves on the accuracy of the first decider [Figs. 5(a) and 5(b)]. The additional time required to obtain these additional opinions is appreciable and roughly independent of the population size, N [Fig. 5(c)]. Hence, even weak correlations in evidence impact the accuracy of collective decisions.

## IV. DISCUSSION

Humans and other animals integrate evidence to make decisions. Often members of a group or community are faced with the same choices and will use evidence that is available to all of them to decide between a common set of options [43–45]. We have shown that when some observations are made in common, even when no social information is exchanged, the first individual to decide makes the least accurate decision. The accuracy of subsequent decisions increases in the order in which they are made, with few exceptions.

We have focused on agents deciding between two options, so that response accuracy can be computed as exit probabilities of populations of univariate stochastic processes driven by common and independent noise [46]. The accuracy of the first agent to make a decision depends nonmonotonically on the probability c of making a common measurement. When the accuracy of the first decision is at a minimum, roughly half the observations are common. The remaining independent observations allow the agent’s beliefs to diverge, leading the first agent to often choose differently than later deciders.

A similar result holds for groups of observers who have a common initial bias and integrate independent evidence [22]. If there are many such agents, the first decision will almost always correspond to the decision boundary closest to the initial belief [37] and is thus wholly determined by the initial common bias.

We made the simplifying assumption that all agents either jointly make a common observation or all make private observations on each time step. This requires a coordinated measurement process, which is counter to our assumption that agents do not share social information. We could relax this assumption and allow agents to each independently make measurements from two sources, one common to the group and one available only to the agent. With two agents this model is equivalent to the model we analyzed. More generally, different subsets of agents could have access to separate sources of shared information, rather than a single common source available to the entire community. The analysis of these cases becomes more cumbersome, but we expect that our general conclusions will hold.

Agents could also share their decisions, in which case the fact that no decision has been announced up to a time t can be informative. The first agent to reach threshold will know that no other decisions have been made yet. This reveals that other agents have gathered independent evidence that disagrees with the first agent’s choice. The first agent can take this information into account reducing their belief that what they thought is the correct choice holds true. Similar reasoning can lead to intricate social information exchanges [22]. However, humans frequently exhibit correlation neglect [47]. If observers assume that information is uncorrelated, then the model we described here may be applicable even when they observe each other’s decisions.

We have assumed that the agents in the population are identical. If agents have different decision thresholds, early decisions tend to be driven by less evidence [2], generating a decrease in accuracy unrelated to the effect of common observations. Correlated evidence could exacerbate this decrease in accuracy. However, if agents have access to information of different quality, early deciders tend to be those with access to the best information [27]. In this case early decisions can be more accurate than later ones. We expect correlated evidence to still impact the accuracy of the first decision, but the specifics would depend on the quality of common and private evidence.

Except for limiting cases, we found it quite cumbersome to obtain analytical expressions for the accuracy of the first decider and other statistics of the agents’ decisions. However, prior work has shown that the correlated drift diffusion model generated in the macroscopic limit can be solved explicitly using method of images solutions for specific threshold values [35]. In our case thresholds always form a square domain encompassing both agents’ beliefs for N=2 or cubes or hypercubes for N>2, but method of images approach may still be applicable.

Like other mathematical models of cognition, our model only roughly approximates decision-making processes used by humans and animals. Despite its limitations, we believe that our analysis offers important insights independent of the exact way evidence is integrated and decisions formed in groups: Common observations drive the beliefs of individuals in the community in the same direction. If those common observations are misleading, it takes time for private evidence to counter their effect. When deciders use a substantial fraction of common observations to make their decisions, early decisions are most likely consistent with common observations. Thus, if common observations are right (wrong), the first decision tends to be as well. First decisions thus tend to be based predominantly on common evidence, which offers less information than what is implied by the decision threshold. We expect that the resulting asymmetric weight of common evidence in determining the first decision leads to similar effects more generally, e.g., when the population is heterogeneous, faced with more than two choices, or when observations are made asynchronously. Social information exchange would lead to more subtle effects, modulating the impact of common measurements. We have thus described a general mechanism that can affect group decision-making, with implications that transcend specific scenarios. The insights we provided can describe decision-making processes across a range of contexts and could be used to organize and guide more effective individual and group choices.

## APPENDIX A: BELIEFS EVOLVING ON THE INTEGER LATTICE

In the simplest case we can assume that an observer can make only two measurements, $\xi^{+}$ and $\xi^{-}$. We let $P\left(\xi^{\pm }\left|H^{\pm }\right.\right)=p_{+}$ and $P\left(\xi^{\pm }\left|H^{\mp }\right.\right)=p_{-}$ with $p_{+}+p_{-}=1$ and $p_{-}<p_{+}$. Assuming $p_{-}=p_{+}/e$, gives $p_{+}+p_{+}/e=1$ so $p_{+}=e/\left(1+e\right)$, $p_{-}=1/\left(1+e\right)$, and hence, $\text{LLR}\left(\xi^{\pm }\right)=\pm 1$. Binarized evidence samples $\xi^{\pm }$ then increment or decrement each agent’s belief $y_{t}^{i}$ by one, so the sum of an even (odd) number of odd numbers, ±1, will be even (odd). In particular, when $p_{-}=p_{+}/a$ the information provided by observation $\xi^{\pm }$ equals ±lna. As a result, the belief of each agent, $y_{i}$, lies on a lattice defined by ${\left\{n\ln\;a\right\}}_{n\in \mathbb{Z}}$, and we can use the mapping n→nlna or a logarithm in base a to place beliefs on an integer lattice. In the double limit of infinitesimal evidence, $\lim_{a\to 1^{+}}\ln\;a$, and infinitesimal time between observations, we can recover a continuous-time model.

## APPENDIX B: ALTERNATIVE DEFINITIONS OF THE FIRST DECIDER

In the main text we defined the “first decider” as an agent chosen with equal probability from the set of all agents who reach threshold at the same time. Alternatively, we could pool all first deciders across trials and compute the probability an agent in this entire pool makes a correct choice. In the scaling limit, the probability that multiple agents reach the threshold at the same time converges to zero, and the two definitions are equivalent. However, when evidence increments are finite, multiple agents can decide first simultaneously. In that case choosing the first decider within a trial and pooling across trials gives different results.

## APPENDIX C: DERIVATION OF THE SCALING LIMIT

Let $f_{+}\left(\xi \right)$ be a probability distribution of observations, ξ, over an arbitrary set Ξ obtained in state $H^{+}$, and $f_{-}\left(\xi \right)$ the probability distribution of observations over that same set Ξ in state $H^{-}$. Note if the sets of observations for either state differ, then there will be infinitely informative observations which, when observed, would immediately make an agent certain of the state. However, these occurrences could be rare, in which case an accumulation process would still be needed. As previously, we use y to denote accumulated LLR so that in the discrete case we have

(C1)
$$y\left(t\right)=\sum_{s⩽t}\text{LLR}\left(\xi_{s}\right),$$

where $\xi_{s}$ is the observation obtained at time s⩽t. Similarly, in the continuous case

(C2)
$$y\left(t\right)=\int_{0}^{t}\frac{dy\left(s\right)}{ds}ds,$$

where $\frac{dy}{ds}$ is given by the stochastic drift-diffusion equation described previously.

We again assume that in a group of N observers each observer at each time step t makes an independent private observation with probability 1−c, and all observers make a common observation with probability c. Private and common observations have the same conditional distributions, $f_{\pm }\left(\xi \right)$ given the state $H^{\pm }$.

For observations drawn from such general likelihood functions, we can determine the statistics of the limiting stochastic accumulation process by averaging the impact of multiple “subobservations” on short intervals which we shrink to be infinitesimal. Focusing on a single observer i, define a family of stochastic processes parameterized by k, the number of subobservations made in an interval of length Δt. Thus, we expect the LLR increment obtained each Δt is given

$$\begin{array}{l} \text{Δ}y_{t}=\sum_{l=1}^{k}\log\frac{f_{+}\left(\xi_{i,t}^{l}\right)}{f_{-}\left(\xi_{i,t}^{l}\right)}\\ \;\;\;\;=E_{\xi }\left[\sum_{l=1}^{k}\log\left.\frac{f_{+}\left(\xi_{i,t}^{l}\right)}{f_{-}\left(\xi_{i,t}^{l}\right)}\right|H\right]\\ \;\;\;\;+\left(\sum_{l=1}^{k}\log\frac{f_{+}\left(\xi_{i,t}^{l}\right)}{f_{-}\left(\xi_{i,t}^{l}\right)}-E_{\xi }\left[\sum_{l=1}^{k}\log\left.\frac{f_{+}\left(\xi_{i,t}^{l}\right)}{f_{-}\left(\xi_{i,t}^{l}\right)}\right|H\right]\right). \end{array}$$

We can split the sum not contained in an expectation into those observations drawn from the common pool and those not,

$$\text{Δ}y_{t}=E_{\xi }\left[\sum_{l=1}^{k}\log\left.\frac{f_{+}\left(\xi_{i,t}^{l}\right)}{f_{-}\left(\xi_{i,t}^{l}\right)}\right|H\right]+\left(\sum_{l=1}^{k_{c}}\log\frac{f_{+}\left(\xi_{i,t}^{l,c}\right)}{f_{-}\left(\xi_{i,t}^{l,c}\right)}+\sum_{l=1}^{k+k_{c}}\log\frac{f_{+}\left(\xi_{i,t}^{l,n}\right)}{f_{-}\left(\xi_{i,t}^{l,n}\right)}-E_{\xi }\left[\sum_{l=1}^{k}\log\left.\frac{f_{+}\left(\xi_{i,t}^{l}\right)}{f_{-}\left(\xi_{i,t}^{l}\right)}\right|H\right]\right),$$

where $\xi_{i,t}^{l,c}$ are samples the ith agent sees from the common pool and $\xi_{i,t}^{l,n}$ are those they see from the independent pool. For large k while keeping Δt fixed, we know the number of common observations will scale as $k_{c}\approx ck$, so assigning

$$\pm \mu \text{Δ}t\equiv E_{\xi }\left[\sum_{l=1}^{k}\log\left.\frac{f_{+}\left(\xi_{i,t}^{l}\right)}{f_{-}\left(\xi_{i,t}^{l}\right)}\right|H=H^{\pm }\right],$$

assuming $f_{\pm }\left(\xi \right)$ are scaled appropriately as Δt→0. We then estimate the variability in the incremental process as k→∞ by computing

$$\begin{array}{l} \langle {\left(\sum_{l=1}^{k_{c}}\log\frac{f_{+}\left(\xi_{i,t}^{l,c}\right)}{f_{-}\left(\xi_{i,t}^{l,c}\right)}+\sum_{l=1}^{k-k_{c}}\log\frac{f_{+}\left(\xi_{i,t}^{l,n}\right)}{f_{-}\left(\xi_{i,t}^{l,n}\right)}-E_{\xi }\left[\sum_{l=1}^{k}\log\left.\frac{f_{+}\left(\xi_{i,t}^{l}\right)}{f_{-}\left(\xi_{i,t}^{l}\right)}\right|H\right]\right)}^{2}\rangle \\ \;\;\;\;\;=\langle \sum_{l=1}^{k_{c}}{\left[\log\frac{f_{+}\left(\xi_{i,t}^{l,c}\right)}{f_{-}\left(\xi_{i,t}^{l,c}\right)}\right]}^{2}\rangle -c\mu^{2}\text{Δ}t^{2}+\langle \sum_{l=1}^{k-k_{c}}{\left[\log\frac{f_{+}\left(\xi_{i,t}^{l,n}\right)}{f_{-}\left(\xi_{i,t}^{l,n}\right)}\right]}^{2}\rangle -\left(1-c\right)\mu^{2}\cdot \text{Δ}t^{2}\\ \;\;\;\;\;=cVar\left[\sum_{l=1}^{k}\log\frac{f_{+}\left(\xi_{i,t}^{l,c}\right)}{f_{-}\left(\xi_{i,t}^{l,c}\right)}\right]+\left(1-c\right)Var\left[\sum_{l=1}^{k}\log\frac{f_{+}\left(\xi_{i,t}^{l,n}\right)}{f_{-}\left(\xi_{i,t}^{l,n}\right)}\right]. \end{array}$$

We can thus approximate the update in the limit of rapid and infinitesimally weak observations using the Donsker Invariance Principle [34]

$$\text{Δ}y_{i,t}\approx \pm \mu \text{Δ}t+\sqrt{\text{Δ}t}\left(\rho_{1-c,\text{Δ}t}\left(t\right)\eta_{1-c}+\rho_{c,\text{Δ}t}\left(t\right)\eta_{c}\right),$$

where $\eta_{c}$ and $\eta_{1-c}$ are random variables with standard normal distributions, and

(C3)
$$\begin{array}{l} \;\;\;\;\;\;\pm \mu =\frac{1}{\text{Δ}t}E_{\xi }\left[\left.\log\frac{f_{+}\left(\xi_{i,t}\right)}{f_{-}\left(\xi_{i,t}\right)}\right|H^{\pm }\right],\\ \rho_{1-c,\text{Δ}t}^{2}\left(t\right)=\frac{\left(1-c\right)}{\text{Δ}t}Var_{\xi }\left[\left.\log\frac{f_{+}\left(\xi_{i,t}\right)}{f_{-}\left(\xi_{i,t}\right)}\right|H^{\pm }\right],\\ \;\;\;\rho_{c,\text{Δ}t}^{2}\left(t\right)=\frac{c}{\text{Δ}t}Var_{\xi }\left[\left.\log\frac{f_{+}\left(\xi_{i,t}\right)}{f_{-}\left(\xi_{i,t}\right)}\right|H^{\pm }\right]. \end{array}$$

The drift $h_{\text{Δ}t}$ and the variances $\rho_{c,\text{Δ}t}^{2}$, $\rho_{1-c,\text{Δ}t}^{2}$ will diverge unless $f_{\pm }\left(\xi \right)$ are properly scaled in the Δt→0limit.

Taking Δt→0 gives

(C4)
$$dy=\pm \mu dt+\rho_{1-c}dW_{i}+\rho_{c}dW_{c},$$

where

$$\begin{array}{l} \;\;\;\;\;\pm \mu =\underset{\text{Δ}t\to 0}{\lim}h_{\text{Δ}t}\left(t\right)=E_{\xi }\left[\left.\log\frac{f_{+}\left(\xi \right)}{f_{-}\left(\xi \right)}\right|H^{\pm }\right],\\ \;\;\;\rho_{c}^{2}\left(t\right)=\underset{\text{Δ}t\to 0}{\lim}\rho_{c,\text{Δ}t}^{2}\left(t\right)=cVar_{\xi }\left[\left.\log\frac{f_{+}\left(\xi \right)}{f_{-}\left(\xi \right)}\right|H^{\pm }\right],\\ \rho_{1-c}^{2}\left(t\right)=\underset{\text{Δ}t\to 0}{\lim}\rho_{c,\text{Δ}t}^{2}\left(t\right)=\left(1-c\right)Var_{\xi }\left[\left.\log\frac{f_{+}\left(\xi \right)}{f_{-}\left(\xi \right)}\right|H^{\pm }\right]. \end{array}$$

We note that $dW_{i}$ corresponds to private noise, which is generated independently for each agent. The term $dW_{c}$ is common to all agents.

## APPENDIX D: EXTENDING THE ANALYSIS OF THE DISCRETE MODEL TO MORE THAN TWO AGENTS

Accuracy of the first decider dips even lower when considering more than two agents N>2 [see Fig. 2(b)]. To explain this more general observation, we extend our two-agent analysis. We denote the decision of agent $j\in \left\{1,\ldots ,N\right\}$ by $d_{j}$ and the corresponding decision time by $T_{j}$. The probability that the first decider chooses $H^{+}$ conditioned on the true state is given by

$$P^{\pm }\left(d_{FD}=H^{+}\right)=\sum_{j=1}^{N}P^{\pm }\left(FD=j\left|d_{j}=H^{+}\right.\right)P^{\pm }\left(d_{j}=H^{+}\right).$$

Leveraging exchange symmetry of distinct agents and defining $T=\left(T_{1},\ldots ,T_{N}\right)\in {\mathbb{N}}^{N}$ (the vector of decision times) and $T=\min_{j}T_{j}$ (the time of the first decision), then

(D1)
$$\begin{array}{l} P^{\pm }\left(d_{FD}=H^{+}\right)=NP^{\pm }\left(d_{1}=H^{+}\right)\sum_{t\in {\mathbb{N}}^{N}}P^{\pm }(FD=1\left|\text{T}=t\right.,\\ \;\;\;\;d_{1}=H^{+})P^{\pm }\left(\text{T}=t\left|d_{1}=H^{+}\right.\right), \end{array}$$

where the first term in the sum vanishes if $t_{1}>\min_{1⩽j⩽N}t_{j}$. On the other hand, if $t_{1}=\min_{1⩽j⩽N}t_{j}$, the conditional probability that agent 1 is chosen as the first decider depends on the number of indices j for which $t_{j}=t_{1}$, i.e., the number of agents who simultaneously decide at the time of the first decision. Let $n_{FD}\left(t\right)$ denote the number of these first deciders. Overall, we have

$$\begin{array}{l} P^{\pm }\left(FD=1\left|\text{T}=t,d_{1}=H^{+}\right.\right)\\ \;\;\;\;\;\;\;\;=\left\{\begin{array}{cc} 0, & t_{1}>\min_{1⩽j⩽N}t_{j},\\ 1/n_{FD}\left(t\right), & t_{1}=\min_{1⩽j⩽N}t_{j}. \end{array}\right. \end{array}$$

Thus, we can turn the second term within the sum from Eq. (D1) into an additional sum over the count of agents deciding at the first decision time:

$$\begin{array}{l} P^{\pm }\left(d_{FD}=H^{+}\right)=NP^{\pm }\left(d_{1}=H^{+}\right)\sum_{t_{1}\in \mathbb{N}}\sum_{k=1}^{N}\frac{1}{k}P^{\pm }\\ \;\;\;\times \left(t_{1}={\text{T}}_{1}=T,n_{FD}\left(T\right)=k\left|d_{1}=H^{+}\right.\right). \end{array}$$

As before, we write the LLR as a sum of two terms, one given by the LLR of a randomly selected agent (agent 1) choosing $H^{+}$, $\text{LLR}\left(d_{1}=H^{+}\right)=\log\left[P^{+}\left(d_{1}=H^{+}\right)\right]/P^{-}\left(d_{1}=H^{+}\right)=\theta$, and a second term involving conditional probabilities that the randomly selected agent is the first decider,

$$\begin{array}{l} \text{LLR}\left(d_{FD}=H^{+}\right)=\text{LLR}\left(d_{1}=H^{+}\right)\\ \;\;\;\;\;\;\;\;\;\;\;\;\;\;\;\;\;\;+\text{LLR}\left(FD=1\left|d_{1}=H^{+}\right.\right), \end{array}$$

where

$$\text{LLR}\left(FD=1\left|d_{1}=H^{+}\right.\right)=\log\frac{\sum_{t_{1}\in \mathbb{N}}\sum_{k=1}^{N}\frac{1}{k}P^{+}\left(t_{1}=T_{1}=T,n_{FD}\left(\text{T}\right)=k\left|d_{1}=H^{+}\right.\right)}{\sum_{t_{1}\in \mathbb{N}}\sum_{k=1}^{N}\frac{1}{k}P^{-}\left(t_{1}=T_{1}=T,n_{FD}\left(\text{T}\right)=k\left|d_{1}=H^{+}\right.\right)}.$$

This term has the same form as in the case of two agents and is negative for 0<c<1 for the same reason: Common observations are likely to be in agreement with the decision of the first decider. However, when the first decider is wrong, independent observations of the other observers are more likely to point away from the first decision threshold than when the first decision is correct. Thus, the first decider is less likely to be correct than a randomly selected agent when 0<c<1, in agreement with simulation results. Moreover, the difference between the numerator and denominator grows with the number of agents, reflecting the additional information provided by having even more undecided agents [Fig. 2(b)]. Other agents will make observations countering the first decision when it is incorrect, and consistent with it when it is correct.
